# OmniCellX: A Versatile and Comprehensive Browser-Based Tool for Single-Cell RNA Sequencing Analysis

**DOI:** 10.3390/biology14101437

**Published:** 2025-10-17

**Authors:** Renwen Long, Tina Suoangbaji, Daniel Wai-Hung Ho

**Affiliations:** 1 State Key Laboratory of Liver Research, The University of Hong Kong, Hong Kong, China; 2 Department of Pathology, School of Clinical Medicine, The University of Hong Kong, Hong Kong, China

**Keywords:** scRNA-seq, analysis tool, visualization tool, integrative analysis, graphical user interface

## Abstract

**Simple Summary:**

Single-cell RNA sequencing (scRNA-seq) is a powerful technology that allows scientists to measure the activity of thousands of genes in individual cells, revealing the differences between them. However, the scRNA-seq data is extremely complex and difficult for many researchers to analyze without advanced computational skills. To solve this problem, we developed OmniCellX, a user-friendly browser-based tool that makes the scRNA-seq analysis accessible to everyone. Our goal is to create a tool that is easy to install and use even for beginners while being powerful enough to handle large datasets containing hundreds of thousands of cells. OmniCellX provides guidance for users throughout the entire analysis process, from initial data processing to identifying cell types, discovering which genes are important, and understanding how cells communicate and change over time. It also provides interactive, high-quality plots for exploring results. By simplifying the analysis, OmniCellX empowers researchers to make new discoveries about how cells work in different biological scenarios, ultimately accelerating progress in biomedical research and the development of new treatments.

**Abstract:**

Single-cell RNA sequencing (scRNA-seq) has revolutionized genomic investigations by enabling the exploration of gene expression heterogeneity at the individual cell level. However, the complexity of scRNA-seq data analysis remains a challenge for many researchers. Here, we present OmniCellX, a browser-based tool designed to simplify and streamline scRNA-seq data analysis while addressing key challenges in accessibility, scalability, and usability. OmniCellX features a Docker-based installation, minimizing technical barriers and ensuring rapid deployment on local machines or clusters. Its dual-mode operation (analysis and visualization) integrates a comprehensive suite of analytical tools for tasks such as preprocessing, dimensionality reduction, clustering, differential expression, functional enrichment, cell–cell communication, and trajectory inference on raw data while enabling alternative interactive and publication-quality visualizations on pre-analyzed data. Supporting multiple input formats and leveraging the memory-efficient data structure for scalability, OmniCellX can efficiently handle datasets spanning millions of cells. The platform emphasizes user flexibility, offering adjustable parameters for real-time fine-tuning, alongside extensive documentation to guide users at even beginner levels. OmniCellX combines an intuitive interface with robust analytical power to perform single-cell data analysis and empower researchers to uncover biological insights with ease. Its scalability and versatility make it a valuable tool for advancing discoveries in cellular heterogeneity and biomedical research.

## 1. Introduction

Individual cells have unique expression characteristics when examined closely, and the developmental or disease onset trajectory depends on the right genes being switched on or off at the right time. The difference in gene transcription between individual cells or groups of cells would be overlooked in bulk-cell RNA sequencing, and the advent of single-cell RNA sequencing (scRNA-seq) technology has made it possible to look at the unique gene signatures of individual cells. scRNA-seq can systematically profile the expression levels of mRNA transcripts for each gene at single-cell resolution. It enables a greater understanding of cellular diversity and heterogeneity, variation between individuals, and identification of rare cell types.

With the rapid advancement and reduced cost of scRNA-seq [1], this technology has become a powerful method for quantifying key biological processes, leading to an exponential growth in related publications and publicly available datasets. While this abundance of data enables broad exploration, it also creates a significant analytical challenge, as integrating large datasets demands robust computational tools. A typical scRNA-seq analysis involves multiple steps; each requires a different software package [2], such as Seurat (v5) [3] for general analysis or CellPhoneDB (5.0.1) [4] for studying cell–cell interactions. However, most existing tools are command-line-based, requiring substantial programming skills and computational expertise. More importantly, there are numerous tools capable of performing tasks throughout the scRNA-seq analysis process, each with its own characteristics, advantages, and limitations [5,6,7,8]. There are overwhelming choices of tools for different dedicated steps, but integrating them into a complete and practical analysis pipeline/workflow could be technically challenging. This technical barrier can be daunting, particularly for wet-lab scientists, and may discourage them from utilizing scRNA-seq in their study.

In this study, we developed OmniCellX, an intuitive browser-based application for real-time scRNA-seq data analysis and visualization. More importantly, OmniCellX offers a complete analysis pipeline for scRNA-seq data of different platforms. Furthermore, it allows flexibility by interactively accepting user-defined parameters throughout the whole analysis process. All in all, this user-friendly browser-based interface simplifies the analysis process and enables scientists to focus on biological insights rather than computational complexity.

## 2. Methods

### 2.1. Programming Codes and Docker Image for Installation and Execution

The OmniCellX application is a browser-based all-in-one analytical platform with a core written in Python3 programming language, while the front-end user interface was implemented by lightweight Javascript library and in-house scripts. All the application code, libraries, tools, dependencies and other files necessary to the execution were encapsulated as a Docker image. Users can pull the image and deploy this service on a personal computer or server without prior installation of any development tools. All the end-to-end workflows are accessible using a simple point-and-click graphical user interface (GUI) through web browser after the docker container works successfully. The detailed instructions and codes used in this article are available in GitHub (https://github.com/longrw/OmniCellX, accessed on 1 August 2025). For optimal performance, a minimum computer configuration of 8 CPU cores and 64 GB RAM is recommended to ensure smooth operation and efficient data processing.

**Figure 1:**
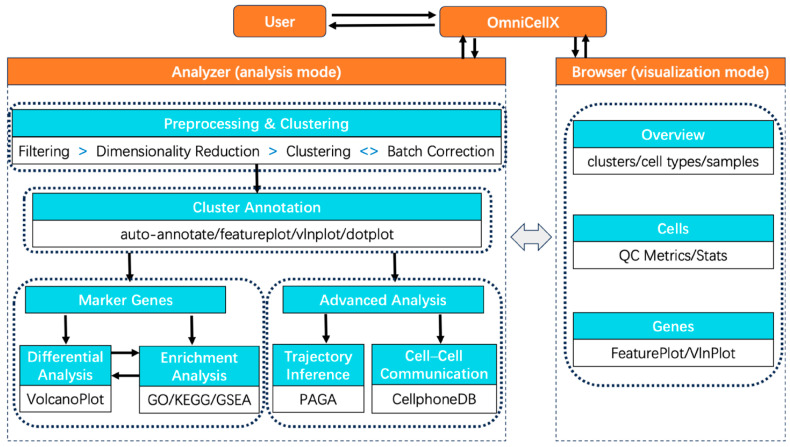
The workflow of OmniCellX. Bashed boxes present all functions involved in two models.

In general, scRNA-seq analysis workflow consists of multiple steps, including reads mapping, generating expression matrix, cell filtering, data normalization, dimensionality reduction, clustering, cell type annotation, differential expression detection, gene-set enrichment analysis, cell–cell communication pseudo-time trajectory inference, etc. The OmniCellX application was designed to offer a complete analysis pipeline of scRNA-seq data after genome alignment. It enables users to seamlessly load gene expression matrix generated from different platforms including 10X Genomics or pre-analyzed data objects. At the initial step, users need to select the mode on the initialization page. The analysis mode contains comprehensive analysis workflow, while the visualization mode aims to allow researchers to directly visualize pre-analyzed results. The workflow of OmniCellX can be viewed in Figure 1.

### 2.2. Basic Analysis Workflow

In the analysis mode, OmniCellX streamlines the analysis workflow from preprocessing to differential analysis. Most popular tools, such as Scanpy (1.11.2), have the common basic analysis workflow, including filtering, dimensionality reduction and clustering. Here, we adopted the basic workflow of Scanpy and incorporated it into the preprocessing and clustering functions of OmniCellX. When creating a new project in this mode, users need to upload sample matrix data for downstream analysis. Results from Cell Ranger on 10X Genomic platform, consisting of barcodes, features and counts files or files in .h5 format are suitable as input. Plain text file (.txt) containing gene expression count matrices with gene annotation as row names and cell barcodes as columns names is also acceptable. All the uploaded files will be automatically identified and loaded into an AnnData [9] object due to its greater memory and computational efficiency. AnnData object can be saved as .h5ad format, which can serve as an input for OmniCellX as well.

Cell filtering can ensure that only high-quality data proceeds to downstream analysis. During filtering, users are allowed to define multiple cut-off values, such as minimum number of detected genes and cells, and maximum percentage of mitochondrial genes. Together with the visualization plots of these metrics, users can evaluate the effect of filtering and adjust the quality thresholds if needed. After quality control, dimensionality reduction should be carried out because of the high dimensionality of scRNA-seq data. Principal Component Analysis (PCA) is used to summarize the genes of the first N principal components, while non-linear dimensional reduction techniques, such as Uniform Manifold Approximation and Projection (UMAP) and t-distributed Stochastic Neighbor Embedding (t-SNE), are also performed in order to visualize and explore these datasets from high-dimensional to two-dimensional space. As a next step, cell clustering aims to group cells into clusters according to the similarity (or distance) of gene expression patterns in cells. The Leiden [10] algorithm is offered as a default option to cluster the cells in OmniCellX. With the resolution parameter, users can set the granularity of the downstream clustering, providing flexibility to explore both broad populations and fine-grained subpopulations. All these clusters would be defined biologically distinct populations in downstream analysis. Additionally, batch effects, often introduced by technical differences in sample preparation, sequencing protocols, or technical artifacts, represent a significant challenge in scRNA-seq data analysis. Correcting for batch effects is essential to ensure that datasets from multiple samples can be integrated seamlessly, enabling robust identification of shared and unique cell populations across conditions or experiments. Harmony [11] is the preference option here to integrate different experiments.

### 2.3. Cluster Annotation

Cluster annotation is a process of labeling groups of cells generated by clustering, which is fundamental and crucial step for researchers to gain insight into biological study in downstream analysis. There are multiple ways to annotate cells in single-cell data, while each of these methods is ultimately based on the expression of specific genes or gene sets, or general transcriptomic similarity between cells. First, we offer the classical way to perform cell type annotation based on a single or small set of marker genes known to be associated with a particular cell type. In OmniCellX, an intuitive interface enables users to define identity, select coloring scheme, perform merging, and re-clustering for the cell clusters. Meanwhile, two commonly used tools, FeaturePlot and VlnPlot, allow users to visualize the expression of marker genes related to specific cell types. Although manual annotation with a predefined set of marker genes is the gold standard method for cell type annotation, a set of tools such as SingleR [12], CellAssign [13], Garnett [14], and CellTypist (1.6.3) [15] have been developed to annotate cells automatically to some extent. Here, we also provide CellTypist (1.6.3) as an alternative way to perform unsupervised in silico cell annotation. Since automated cell annotation tools still have significant limitations and should only be considered as auxiliary instrument for cell typing. Therefore, we believe that automated annotation complemented by classical marker-based annotation method will collectively assist users in completing cell annotation tasks.

### 2.4. Differential Expression Analysis

To find marker genes specifically enriched in cell types or cell clusters, users can perform differential expression analysis for genes between clusters or compare a cell type between groups under study. A differential gene expression test usually returns the log2(fold-change) and the adjusted *p*-value per compared genes per compared conditions. This list can then be displayed as tables and violin plots showing the most differentially expressed genes. The results of the differential analysis can then be used for subsequent enrichment analysis. Differential expression results can be filtered further and visualized with a volcano plot. The list of downregulated or upregulated genes can be used to perform Gene Ontology (GO) [16] or Kyoto Encyclopedia of Genes and Genomes (KEGG) [17] enrichment analysis using EnrichR [18]. Gene Set Enrichment Analysis (GSEA) [19] is another method offered to analyze gene expression differences between biological states/conditions using predefined gene sets.

### 2.5. Other Advanced Analyses

scRNA-seq enables a wide range of advanced analyses, and these analyses provide further insights into cellular heterogeneity, dynamic processes and disease mechanisms, making single-cell technology a powerful tool in biology and medicine studies. OmniCellX currently offers two key advanced analyses, naming trajectory inference and cell–cell communication. Cell trajectory analysis can infer the cell differentiation trajectory or the source of a certain type of cell differentiation during the development process. Here, we use PAGA [20] to perform trajectory analysis of the cells. The starting point (or root) in PAGA trajectory analysis typically corresponds to the most undifferentiated or progenitor cell population from which differentiation proceeds. Users need not define the root and it will be automatically selected using default parameters of PAGA. Cell–cell interaction is another advanced analysis after cluster annotation step. In multicellular organisms, the dynamic coordination of cells is involved in many biological processes, such as triggering of apoptosis and cell migration, and is consequently essential in homeostasis and disease initiation. In the context of cancer, cell–cell communication is pivotal to the interactive dynamics within the complex tumor microenvironment that support various oncogenic processes [21]. OmniCellX offers CellPhoneDB to infer cell–cell communication networks. All these advanced tools are available as optional steps for users.

### 2.6. Data Visualization

Visualization is another critical component of scRNA-seq analysis, as it helps researchers explore complex datasets, identify patterns, and communicate findings effectively. Unlike the analysis mode that emphasizes performing different essential steps for analyzing the dataset, visualization mode is available as an alternative if users have already obtained pre-analyzed data. One scenario could be they received pre-analyzed data from collaborators. In the visualization mode, users are allowed to directly visualize and interact with their own data and results. A wide range of plots are available, each of which is fully customizable to fit their design preference, enabling the generation and export of publishable figures. Through an interactive and intuitive graphical interface, users can overview panels of samples and clusters or explore similarities and heterogeneity between samples and cell clusters in two-dimensional projections such as UMAP or t-SNE. QC metrics and frequency plot for cell set composition are browsed at cells level part, while the expression level of single genes or gene sets would be displayed at genes level by feature plot or violin plot.

## 3. Results

### 3.1. Overview and Key Features of OmniCellX

OmniCellX is designed as a comprehensive single-cell transcriptomic analysis and visualization tool. It is deployed via Docker, allowing users to perform all analyses directly through a web browser and view results in an interactive visualization mode. Notably, OmniCellX offers an advanced cell annotation interface, a critical step in single-cell transcriptomic analysis. Traditionally, even experienced bioinformaticians must repeatedly refine their definition to achieve accurate cell annotation, which is a time-consuming and labor-intensive process. However, with OmniCellX’s user-friendly and automatic cell annotation function, wet-lab scientists can efficiently and accurately annotate cell populations in their data.

### 3.2. Execution of OmniCellX

#### 3.2.1. Clustering and Cell Type Annotation

**Figure 2:**
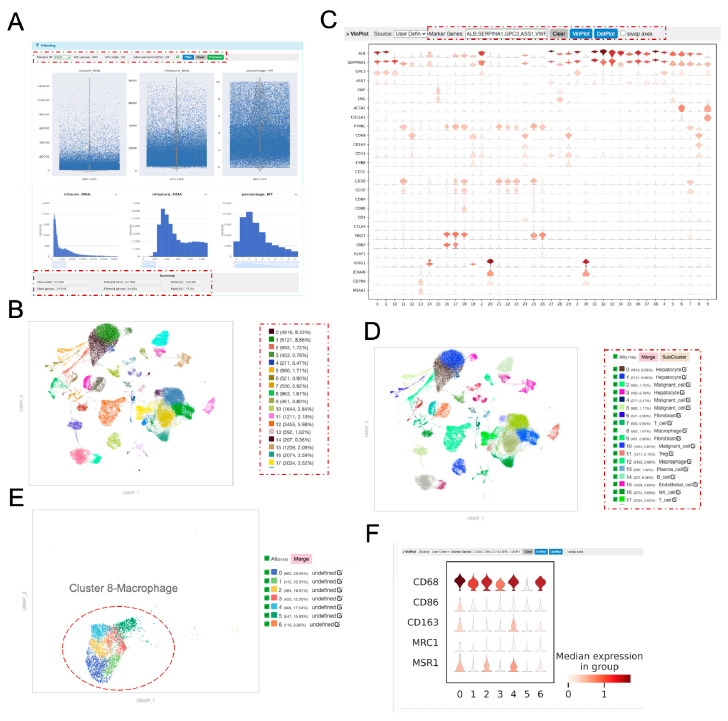
Pre-processing and clustering of single-cell data. (**A**) Violin and bar plots showing the number of cells before and after filtering based on the following criteria: number of counts (>200), number of features (>20), and percentage of mitochondrial RNA (<20%). (**B**) UMAP plot of samples after batch correction, colored by clusters. The legend displays the cell count and proportion of each cluster. (**C**) Violin plots showing the median expression levels of canonical markers in each cluster. (**D**) UMAP plot of major cell types, colored by cell types. The legend includes all clusters and their corresponding cell types. (**E**) Re-clustering UMAP plot of macrophages (from panel D), colored by re-clusters prior to defining subtypes. (**F**) Violin plots showing the expression levels of genes in macrophage sub-clusters.

To demonstrate the functionalities of OmniCellX, we analyzed a single-cell dataset derived from hepatocellular carcinoma (HCC) across several published studies [22,23,24]. In fact, we have conducted internal testing using different publicly available datasets. In the execution, a new project was created, and the data matrices for these samples were uploaded in analysis mode. Following quality control and batch correction (Figure 2A), transcriptomic data for 57,789 cells were obtained. The filtering module displayed the number of cells and genes before and after quality control. Subsequently, 39 cell clusters were identified using the Leiden graph-clustering algorithm with the default resolution (1.0) (Figure 2B). Cluster annotation was performed by examining canonical marker genes of major cell types, supplemented by automatic annotation methods. Based on the expression profiles of canonical markers across the clusters (Figure 2C), nine major cell types [25,26] were identified: T cells, NK cells, B cells, fibroblasts, macrophages, endothelial cells, hepatocytes, and malignant cells (Figure 2D).

#### 3.2.2. Subclustering and Reclustering

OmniCellX also supports further classification of major cell types. For instance, cluster 8, identified as macrophages in the initial analysis, was re-clustered into seven sub-clusters. Examining gene expression patterns within these sub-clusters enabled the identification of additional macrophage subtypes (Figure 2E,F).

#### 3.2.3. Additional Testing by Other Publicly Available Datasets

We additionally applied OmniCellX to multiple datasets. One HCC dataset was assembled by merging samples downloaded from LiverSCA [27,28], comprising more than 130,000 cells (Figure S1). Another dataset consisted of human PBMCs (https://www.10xgenomics.com/datasets/human-pbmc-from-a-healthy-donor-10-k-cells-v-2-2-standard-4-0-0) obtained from 10x Genomics, containing over 10,000 cells (Figure S2). Because the clustering stage is the most time- and memory-intensive step of the workflow, we recorded the runtime and peak memory usage for each dataset during this stage (Table S1). In general, they roughly increased with the size of dataset.

#### 3.2.4. Differential Gene Expression and Gene Set Enrichment Analysis

**Figure 3:**
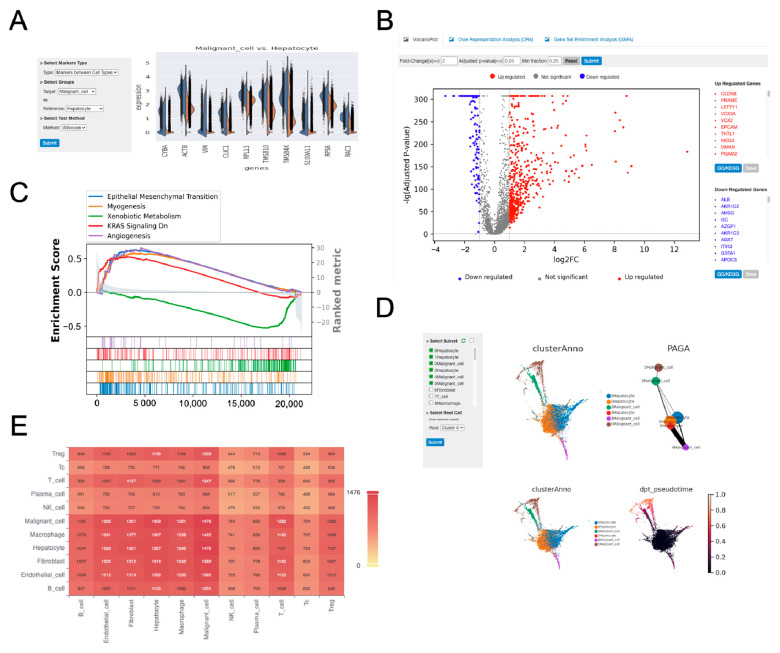
Further comprehensive analysis focusing on malignant cells. (**A**) The top 10 marker genes identified in malignant cells compared to hepatocytes, highlighting genes with significant differential expression. (**B**) Volcano plot showing differentially expressed genes (DEGs) between malignant cells and hepatocytes, with upregulated and downregulated genes marked by significance thresholds. (**C**) Gene Set Enrichment Analysis (GSEA) of DEGs in malignant cells between hepatocytes. (**D**) Pseudo-time trajectory analysis tracing the progression of hepatocytes transitioning into malignant cells, with malignant cells identified as the final state. (**E**) Heatmap illustrating the number of significant cell–cell interactions between each cell type pair.

Using differential expression analysis module, the top 10 marker genes in malignant cells were identified by comparing their expression profiles with those of hepatocytes. The differential expression of these genes was prominently observed in both cell types (Figure 3A). All differentially expressed genes (DEGs) from the comparison are visualized in a volcano plot, with upregulated and downregulated genes listed alongside the plot (Figure 3B). GSEA further revealed that malignant cells exhibited elevated activity in epithelial–mesenchymal transition (EMT) and angiogenesis pathways compared to hepatocytes (Figure 3C). EMT is a process where epithelial cells transition to a mesenchymal phenotype, enhancing their motility and invasiveness, which is crucial for metastasis [29]. Studies have shown that EMT plays a significant role in HCC progression [30], with strong inducers of EMT like transforming growth factor-β (TGF-β) can promote both fibrogenesis and carcinogenesis [19]. The increased angiogenesis observed in malignant cells, facilitated by growth factors such as vascular endothelial growth factor (VEGF), is essential for tumor growth and metastasis, making it a key target for HCC therapies [31,32].

#### 3.2.5. Pseudo-Time Trajectory Analysis and Cell–Cell Communication

Pseudo-time trajectory analysis indicated that malignant cells represented the terminal state of differentiated hepatocytes (Figure 3D). This suggests a potential developmental pathway where hepatocytes transition towards a malignant state over time. Cellular communication analysis showed strong interactions among most cell populations, with the exception of cytotoxic T cells, NK cells, and plasma cells, which exhibited limited interactions (Figure 3E). In order to indicate the generosity of OmniCellX, we complementarily performed the pseudo-time trajectory and cell–cell communication analyses by Monocle [33] and CellChat [34], respectively. Consistent with the findings from OmniCellX, Monocle identified a transition from hepatocytes to malignant cells (Figure S3A), while CellChat revealed diminished interactions between malignant cells and plasma cells (Figure S3B).

### 3.3. Comparison of Functionalities Between OmniCellX and Other Tools

Numerous valuable platforms have been developed to streamline single-cell analysis workflows. Some widely used examples include ASAP [35], Loupe Browser, CellxGene [36] and the UCSC Cell Browser (Table 1). ASAP is a powerful and accessible tool for researchers who need to perform standardized single-cell RNA-seq analysis, particularly for annotating and comparing known datasets. This platform is designed for standardized workflows, which limits its flexibility for users performing highly customized analyses. Loupe Browser is a powerful and user-friendly tool for visualizing and exploring single-cell RNA sequencing data generated using 10X Genomics platforms. Its intuitive interface, interactive visualizations, and seamless integration with 10X data make it an excellent choice for researchers who want to quickly explore their data and generate insights without requiring extensive computational expertise. However, its limitations in terms of data compatibility and customization may make it less suitable for users working with other platforms. More importantly, it is not suitable for studies that require sophisticated analytical capabilities. Both CellxGene and UCSC Cell Browser are primarily developed for data exploration and visualization. They lack comprehensive computational pipelines for tasks like preprocessing, differential expression analysis, or data integration. This means that users may need to rely on other tools for advanced analytical tasks.

In contrast, OmniCellX stands out by integrating a broader range of advantages. One of the standout features of OmniCellX is its Docker-based installation, which significantly reduces the technical barriers associated with setting up bioinformatics tools. This ensures that users can quickly deploy OmniCellX on their local machines or computer clusters without the need for any configurations. The Docker framework also guarantees scalability, enabling OmniCellX to handle both small-scale and large-scale datasets efficiently, making it suitable for a wide range of research projects. Secondly, OmniCellX operates in two distinct modes, analysis mode and visualization mode. The analysis mode integrates a comprehensive suite of single-cell analysis tools and algorithms, allowing users to perform tasks such as dimensionality reduction, clustering, differential expression analysis, cell–cell communication and trajectory inference. The visualization mode, on the other hand, provides interactive and customizable visual representations of the analysis results. This dual-mode approach ensures that users can not only analyze their data but also generate publication-quality figures with adjustable parameters, facilitating seamless data interpretation and dissemination. Thirdly, OmniCellX supports multiple single-cell expression matrix formats as input. This flexibility ensures compatibility with data from different single-cell platform, such as 10x Genomics, Smart-seq2, and others. Fourthly, OmniCellX utilizes AnnData objects, a highly efficient format for storing and analyzing single-cell data, as its data structure owing to its greater memory and computational efficiencies. AnnData object’s memory and computational efficiency, as demonstrated by its adoption in the widely used Scanpy [37] toolkit, enables OmniCellX to handle large datasets (it depends on the available computational resources of the system for its successful execution), making it a robust solution for large-scale studies. Fifthly, OmniCellX also emphasizes user customization and flexibility. The tool provides a wide range of adjustable parameters, allowing users to fine-tune their analyses in real-time and generate results that meet their specific requirements. This iterative approach ensures that researchers can achieve optimal outcomes tailored to their experimental needs. Finally, OmniCellX is supported by comprehensive documentation, which serves as a valuable resource for users at all stages of their analysis. Whether troubleshooting technical issues or exploring advanced analytical functionalities, users can refer to the detailed guides and tutorials to resolve their queries and maximize the utility of the tool.

## 4. Discussion

scRNA-seq has revolutionized the field of single-cell genomics by enabling scientists to explore heterogeneity of gene expression at the individual cell level, but the process can be intractable to some researchers. In this article, we introduced OmniCellX, a browser-based scRNA-seq analysis tool designed to simplify and streamline the process of analyzing single-cell transcriptomic data. OmniCellX addresses many of the challenges faced by researchers in the field, offering a user-friendly, scalable, and efficient platform that caters to users with varying levels of computational expertise. By integrating advanced analytical capabilities with an intuitive web-based interface, OmniCellX empowers researchers to explore the complexity of single-cell data and extract meaningful biological insights easily.

OmniCellX is designed to provide researchers, particularly those without bioinformatics expertise, with a powerful platform for single-cell transcriptomic analysis. There are several limitations that require further development and optimization in our further updates. First, the choices of usable algorithms could be more comprehensive. For example, batch effect correction, trajectory inference and cell–cell communication analysis should incorporate additional algorithms. Second, the advanced single-cell transcriptomic analysis module should extend beyond trajectory inference and cell–cell communication to include transcription factor analysis, copy number variation analysis, etc. We will also add automated multiplet removal in the quality control procedure. To better determine optimal clustering, we will also consider adding cluster stability metrics such as Silhouette Score in the analysis. Additionally, performing integrative investigation using multiple omics data types, such as VDJ-seq and scATAC-seq, will also be a promising direction for OmniCellX’s future development. We are committed to continuously improving and maintaining OmniCellX to better support the research community.

## 5. Conclusions

OmniCellX represents a substantial advancement in the field of single-cell genomics by providing a powerful, accessible, and versatile platform for scRNA-seq data analysis. Its combination of user-friendly design, robust analytical capabilities, and extensive documentation makes it an invaluable resource for researchers aiming to unravel the complexities of cellular heterogeneity and gene expression landscape at the single-cell level. By lowering the barriers to single-cell data analysis, OmniCellX has the potential to accelerate discoveries across a wide range of scientific research areas.

## Figures and Tables

**Table 1:** Comparison of features. √ means include, x means do not include.

Features	OmniCellX	ASAP (v7)	Loupe Browser	CellxGene	UCSC Cell Browser
Type of application	Desktop & Cloud	Cloud	Desktop	Desktop & Cloud	Desktop & Cloud
Multiple inputs supported	√	√	x	√	x
Filtering	√	√	x	√	x
Dimension reduction	√	√	x	√	x
Clustering	√	√	√	√	x
Batch effects removal	√	x	x	x	x
Cluster annotation	√	√	√	√	√
Auto annotation	√	x	x	x	x
Cluster coloring	√	√	√	x	√
Cluster merging	√	x	√	x	√
Subclustering	√	x	√	x	x
Differential expression	√	√	√	√	x
Functional enrichment	√	√	x	x	x
Trajectory inference	√	x	x	x	x
Cell communication	√	x	x	x	x
Gene regulatory network	x	x	x	x	x
QC metrics	√	√	x	x	x
Cell proportion	√	x	x	x	x
Gene distribution	√	x	x	x	x
Pair analysis	√	x	x	√	x
Publication-ready figures	√	√	√	x	√
Detailed documentation	√	√	x	√	√
Multi-omics supported	x	x	√	x	x
Data sharing	√	√	x	x	√

## Data Availability

Project name: OmniCellX; Project home page: https://github.com/longrw/OmniCellX, accessed on 1 August 2025; Operating system(s): Platform independent; Programming language: Python and Javascript; Other requirements: Python 3.8 or higher; License: GNU GPL.

## Supplementary Materials

The following supporting information can be downloaded at: https://www.mdpi.com/article/10.3390/biology14101437/s1, Figure S1: Pre-processing and clustering of single-cell data including more than 130 thousands cell. Figure S2: Pre-processing and clustering of PBMC single-cell data. Figure S3: Re-analysis pseudo-time and cell and cell communication by Monocle and CellChat. Table S1: Comparison of runtime and memory usage for each dataset during the clustering step.
