# Supplementary material for: OmniCellX: A Versatile and Comprehensive Browser-Based Tool for Single-Cell RNA Sequencing Analysis
Source: Biology (Basel). 2025 Oct 17;14(10):1437. doi: 10.3390/biology14101437 (PMC12562230; doi:10.3390/biology14101437)
Supplement: Supplementary file 1 [file biology-14-01437-s001.zip › biology-3867270-supplementary.pdf]

SFigure 1

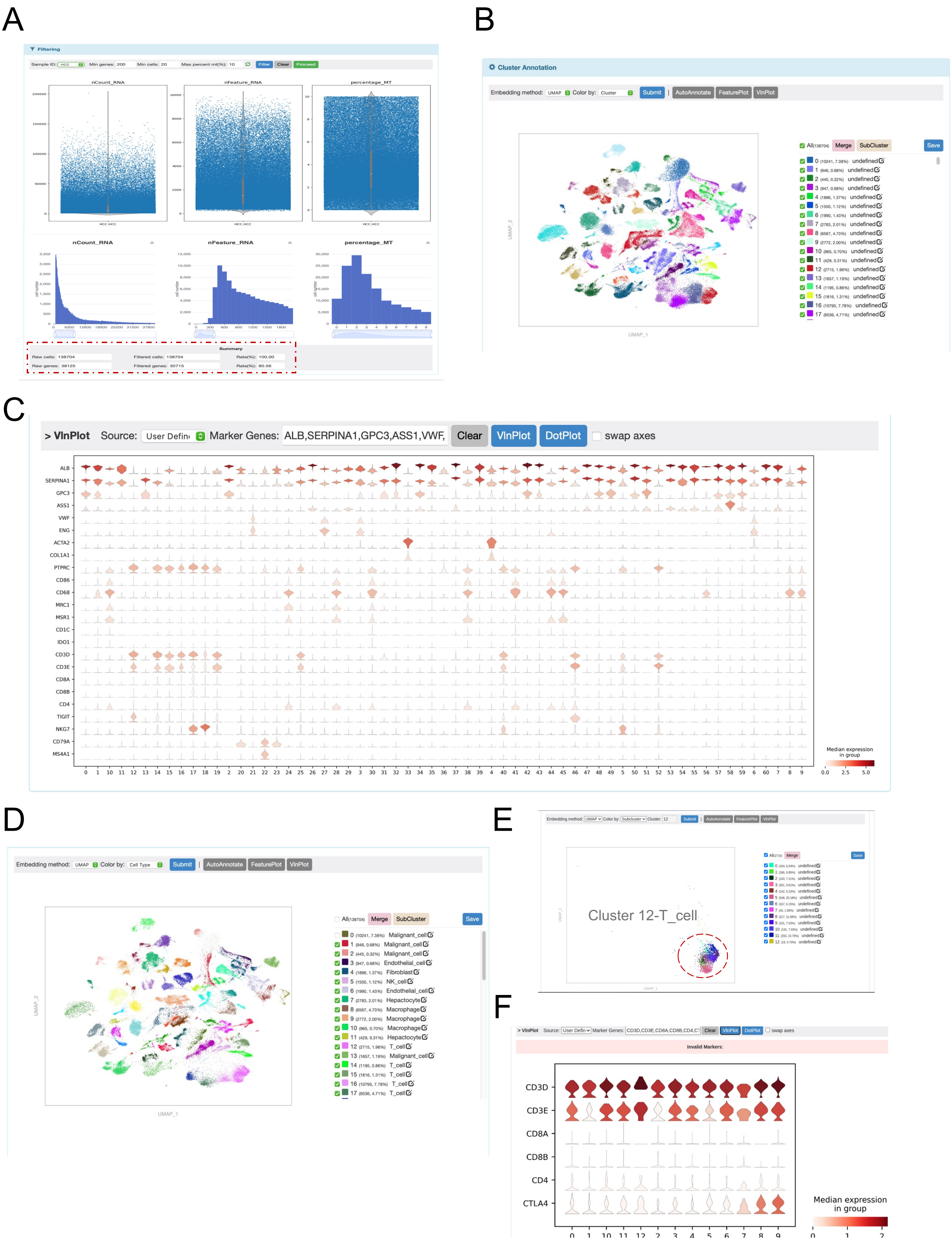

A
B
C
D
E
F
Filtering
Cluster Annotation
Embedding method: UMAP Color by: Cluster Submit | AutoAnnotate FeaturePlot VlnPlot
> VlnPlot Source: User Defin Marker Genes: ALB,SERPINA1,GPC3,ASS1,VWF, Clear VlnPlot DotPlot swap axes
ALB
SERPINA1
GPC3
ASS1
VWF
ENG
ACTA2
COL1A1
PTPRC
CD86
CD68
MRC1
MSR1
CD1C
IDO1
CD3D
CD3E
CD8A
CD8B
CD4
TIGIT
NKG7
CD79A
MS4A1
Median expression in group
Embedding method: UMAP Color by: Cell Type Submit | AutoAnnotate FeaturePlot VlnPlot
Cluster 12-T_cell
Invalid Markers:
CTLA4

SFigure 2

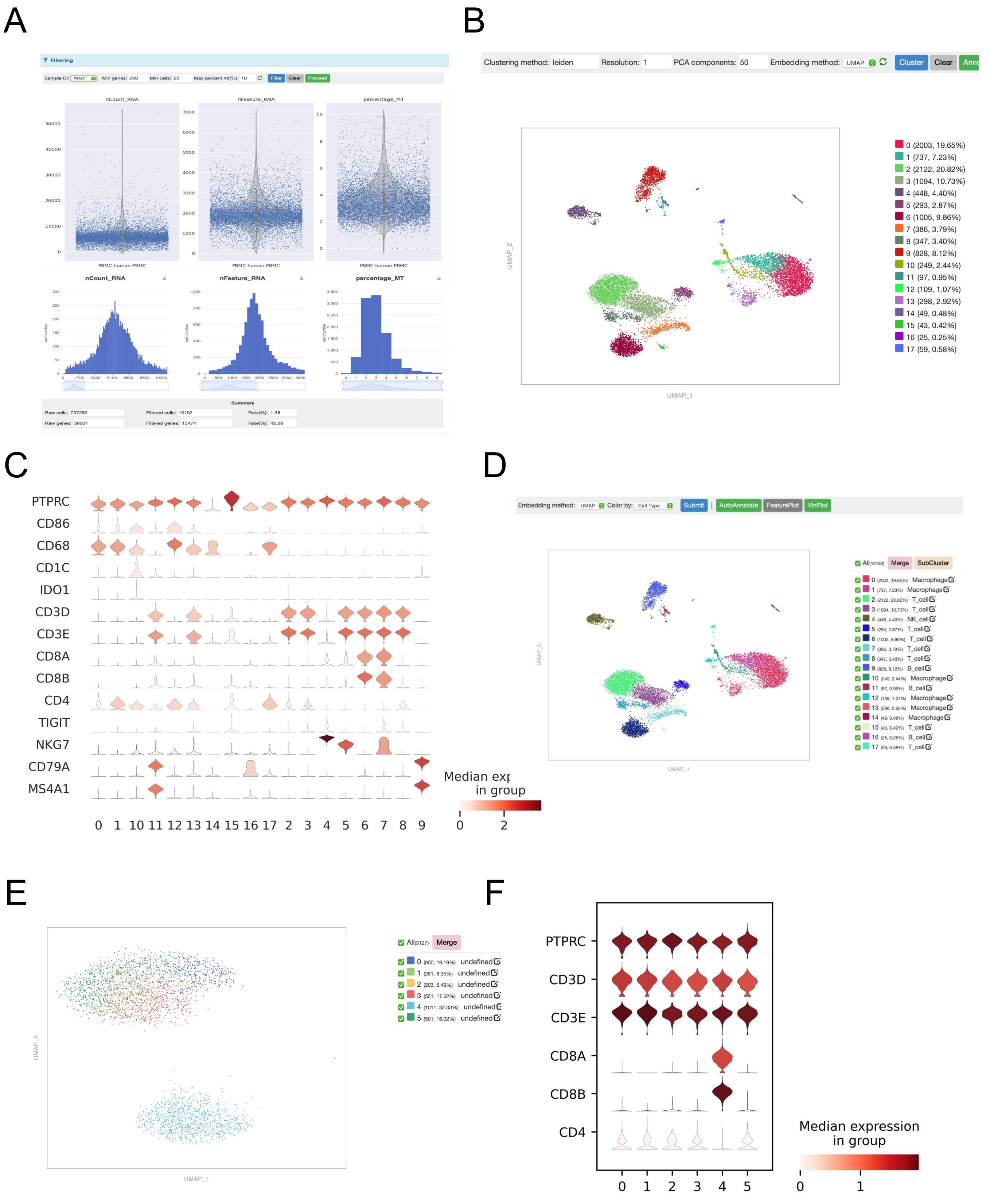
A
B
C
D
E
F
Filtering
nCount_RNA
nFeature_RNA
percentage_MT
PBMC-human.PBMC
Summary
Raw cells: 737280
Filtered cells: 10192
Rate(%): 1.38
Raw genes: 36601
Filtered genes: 15474
Rate(%): 42.28
Clustering method: leiden
Resolution: 1
PCA components: 50
Embedding method: UMAP
Cluster
Clear
UMAP_1
UMAP_2
0 (2003, 19.65%)
1 (737, 7.23%)
2 (2122, 20.82%)
3 (1094, 10.73%)
4 (448, 4.40%)
5 (293, 2.87%)
6 (1005, 9.86%)
7 (386, 3.79%)
8 (347, 3.40%)
9 (828, 8.12%)
10 (249, 2.44%)
11 (97, 0.95%)
12 (109, 1.07%)
13 (298, 2.92%)
14 (49, 0.48%)
15 (43, 0.42%)
16 (25, 0.25%)
17 (59, 0.58%)
PTPRC
CD86
CD68
CD1C
IDO1
CD3D
CD3E
CD8A
CD8B
CD4
TIGIT
NKG7
CD79A
MS4A1
0 1 10 11 12 13 14 15 16 17 2 3 4 5 6 7 8 9
Median exp
in group
Embedding method: UMAP
Color by: Cell Type
Submit
AutoAnnotate
FeaturePlot
VlnPlot
Merge
SubCluster
0 Macrophage
1 Macrophage
2 T_cell
3 T_cell
4 NK_cell
5 T_cell
6 T_cell
7 T_cell
8 T_cell
9 B_cell
10 Macrophage
11 B_cell
12 Macrophage
13 Macrophage
14 Macrophage
15 T_cell
16 B_cell
17 T_cell
0 (600, 19.19%) undefined
1 (261, 8.35%) undefined
2 (203, 6.49%) undefined
3 (551, 17.62%) undefined
4 (1011, 32.33%) undefined
5 (501, 16.02%) undefined
PTPRC
CD3D
CD3E
CD8A
CD8B
CD4
0 1 2 3 4 5
Median expression
in group

SFigure 3

A

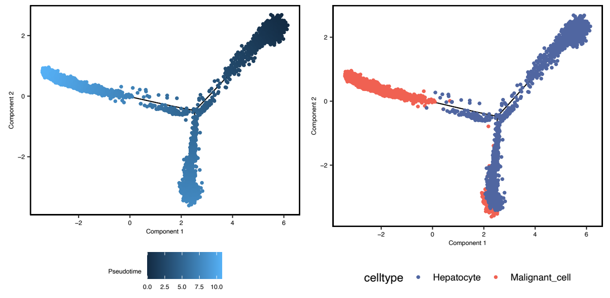
Component 2
Component 1
Pseudotime
0.0 2.5 5.0 7.5 10.0
celltype
Hepatocyte
Malignant_cell

B

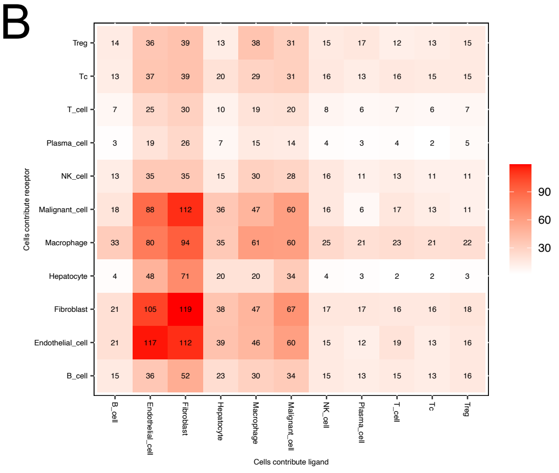
Treg 14 36 39 13 38 31 15 17 12 13 15
Tc 13 37 39 20 29 31 16 13 16 15 15
T_cell 7 25 30 10 19 20 8 6 7 6 7
Plasma_cell 3 19 26 7 15 14 4 3 4 2 5
NK_cell 13 35 35 15 30 28 16 11 13 11 11
Malignant_cell 18 88 112 36 47 60 16 6 17 13 11
Macrophage 33 80 94 35 61 60 25 21 23 21 22
Hepatocyte 4 48 71 20 20 34 4 3 2 2 3
Fibroblast 21 105 119 38 47 67 17 17 16 16 18
Endothelial_cell 21 117 112 39 46 60 15 12 19 13 16
B_cell 15 36 52 23 30 34 15 13 15 13 16
B_cell Endothelial_cell Fibroblast Hepatocyte Macrophage Malignant_cell NK_cell Plasma_cell T_cell Tc Treg
Cells contribute receptor
Cells contribute ligand
90
60
30

Supplementary figure legends

Figure S1. Pre-processing and clustering of single-cell data including more than 130 thousands cell. A. Violin and bar plots showing the number of cells before and after filtering based on the following criteria: number of counts (>200), number of features (>20), and percentage of mitochondrial RNA (<10%). B. UMAP plot of samples after batch correction, colored by clusters. The legend displays the cell count and proportion of each cluster. C. Violin plots showing the median expression levels of canonical markers in each cluster. D. UMAP plot of major cell types, colored by cell types. The legend includes all clusters and their corresponding cell types. E. Re-clustering UMAP plot of T cell (from panel D), colored by re-clusters prior to defining subtypes. F. Violin plots showing the expression levels of genes in T cell sub-clusters.

Figure S2. Pre-processing and clustering of PBMC single-cell data. A. Violin and bar plots showing the number of cells before and after filtering based on the following criteria: number of counts (>200), number of features (>20), and percentage of mitochondrial RNA (<10%). B. UMAP plot of samples after batch correction, colored by clusters. The legend displays the cell count and proportion of each cluster. C. Violin plots showing the median expression levels of canonical markers in each cluster. D. UMAP plot of major cell types, colored by cell types. The legend includes all clusters and their corresponding cell types. E. Re-clustering UMAP plot of T cell (from panel D), colored by re-clusters prior to defining subtypes. F. Violin plots showing the expression levels of genes in T cell sub-clusters.

Figure S3. Re-analysis pseudo-time and cell and cell communication by Monocle and CellChat. A. The pseudo-time trajectory analysis of hepatocytes and malignant cells. B Heatmap plot showed cell cell communication results from CellChat.

Table S1. Comparison of runtime and memory usage for each dataset during the clustering step.

Dataset	CELL COUNT	SOURCE	RUNTIME (S)	MEMORY (GB)
1	138,704	Liver-Human	400	30
2	57,789	Liver-Human	85	16
3	10,192	Blood-Human	43	16
